# Supplementary figures and images for: Specific lid-base contacts in the 26s proteasome control the conformational switching required for substrate degradation
Source: eLife. 2019 Nov 28;8:e49806. doi: 10.7554/eLife.49806 (PMC6910829; doi:10.7554/eLife.49806)

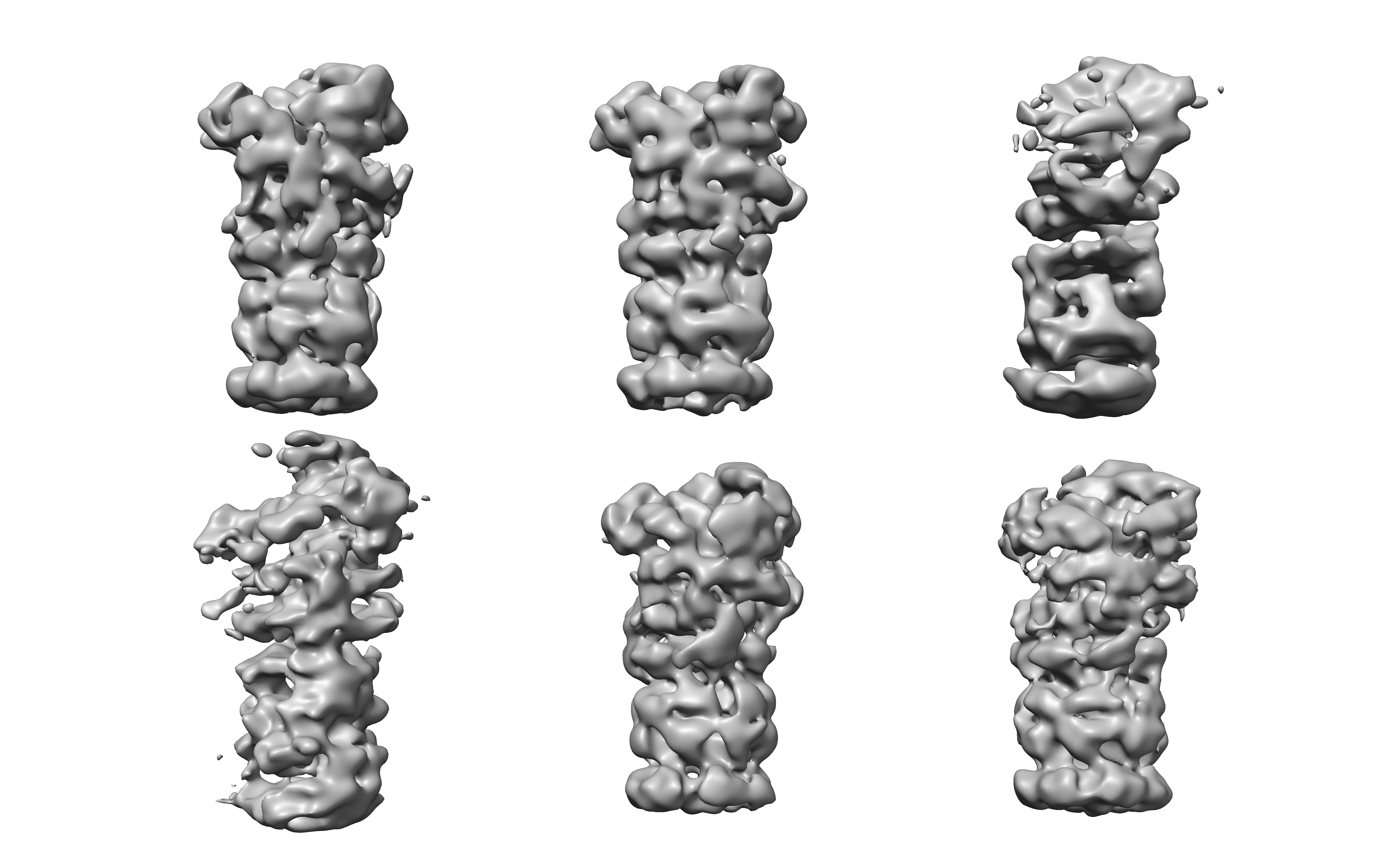

Supplement: Figure 2—source data 1. [file elife-49806-fig2-data1.zip › Figure 2-source data/EM density/MutATP_k6al/image.png]

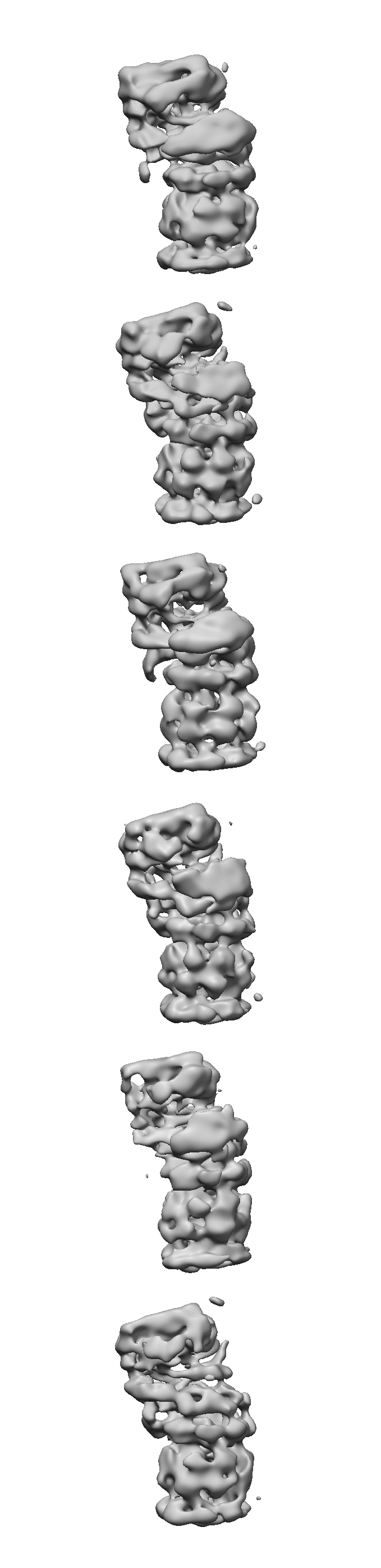

Supplement: Figure 2—source data 1. [file elife-49806-fig2-data1.zip › Figure 2-source data/EM density/WTATP_k6al/tiles_1to6.png]

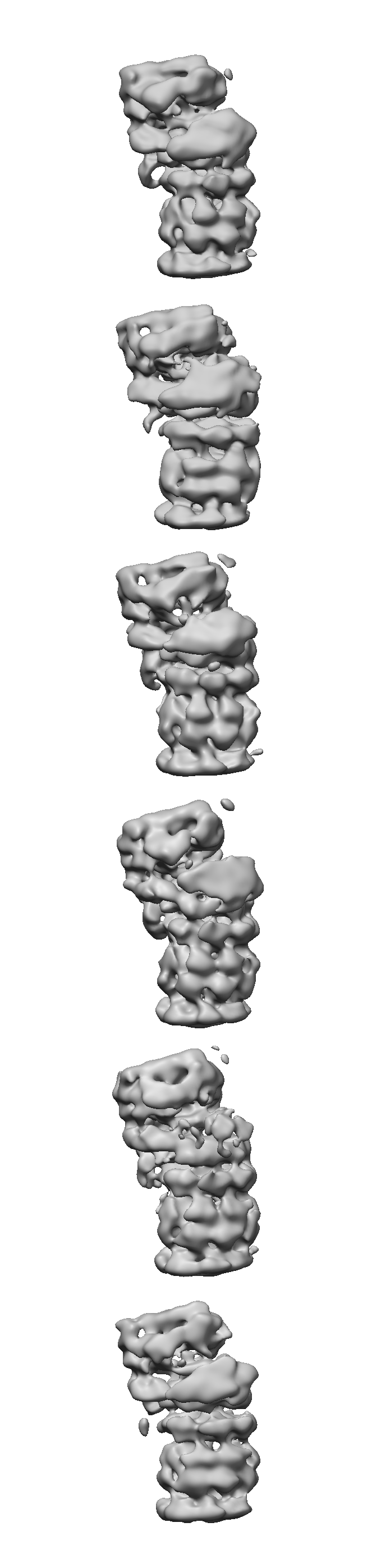

Supplement: Figure 2—source data 1. [file elife-49806-fig2-data1.zip › Figure 2-source data/EM density/MutAtp_recen_k6al/tiles_1to6.png]

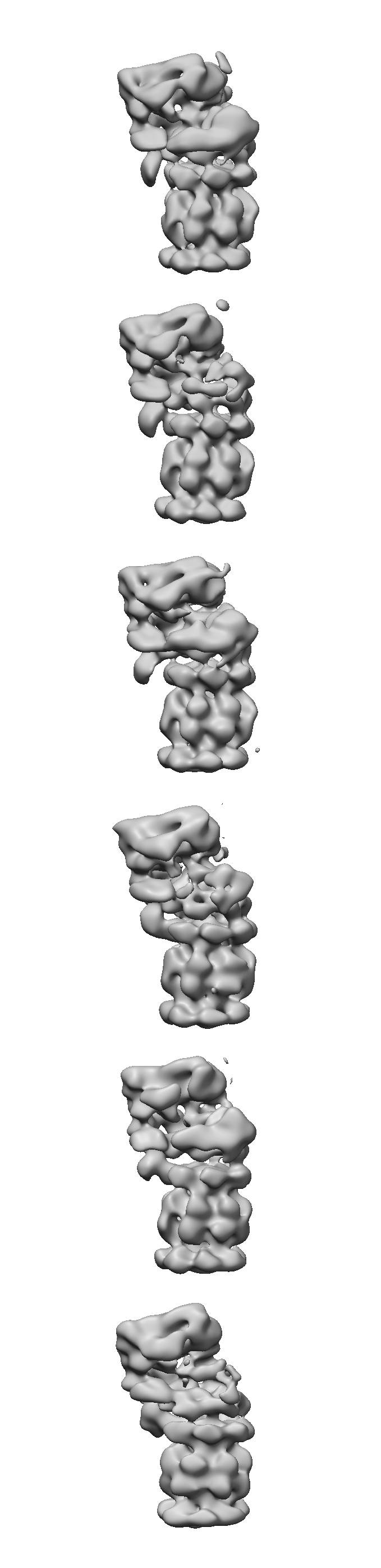

Supplement: Figure 2—source data 1. [file elife-49806-fig2-data1.zip › Figure 2-source data/EM density/MutATPgS_k6al/tiles_1to6.png]

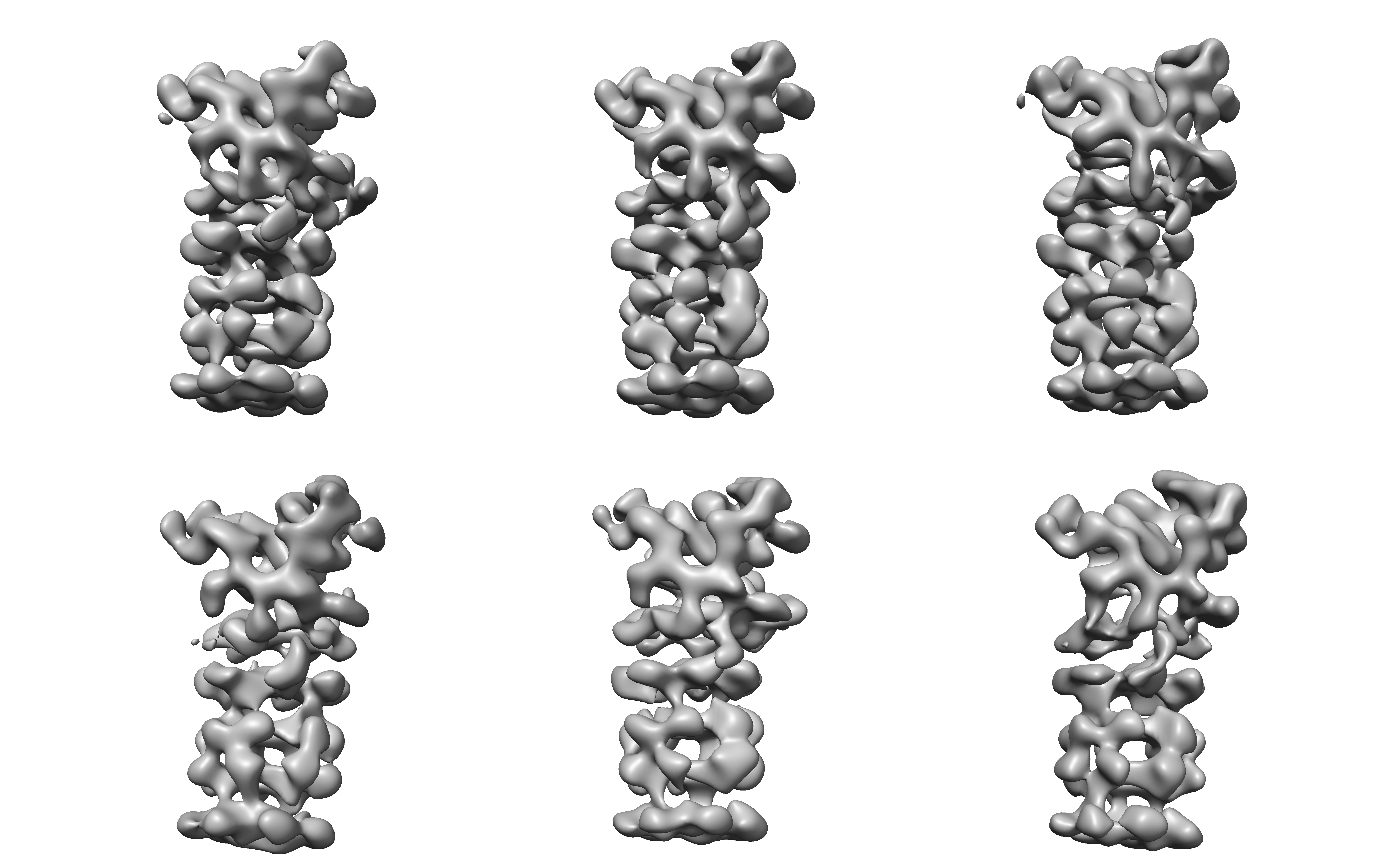

Supplement: Figure 2—source data 1. [file elife-49806-fig2-data1.zip › Figure 2-source data/EM density/MutATPgS_k6al/image.png]

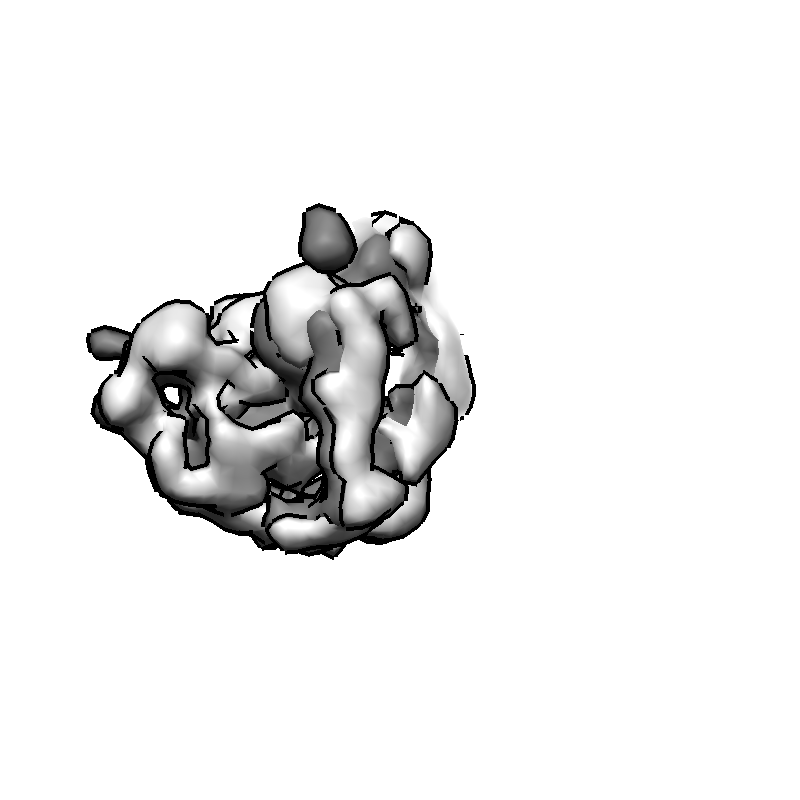

Supplement: Figure 2—source data 1. [file elife-49806-fig2-data1.zip › Figure 2-source data/EM density/WTATPgS_k6al/s2_WA6a.png]

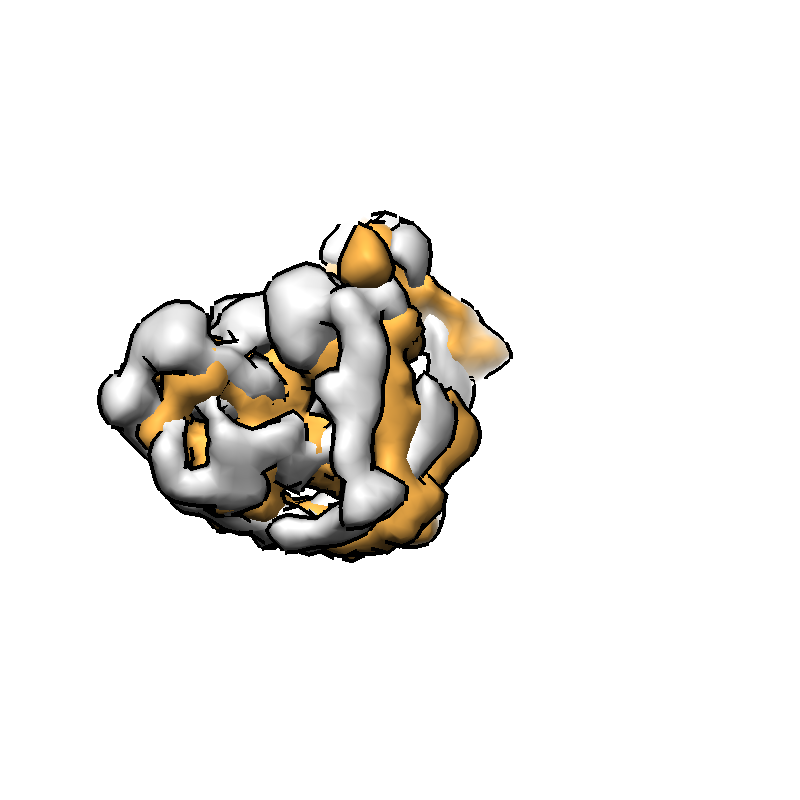

Supplement: Figure 2—source data 1. [file elife-49806-fig2-data1.zip › Figure 2-source data/EM density/WTATPgS_k6al/s3_WA6a.png]

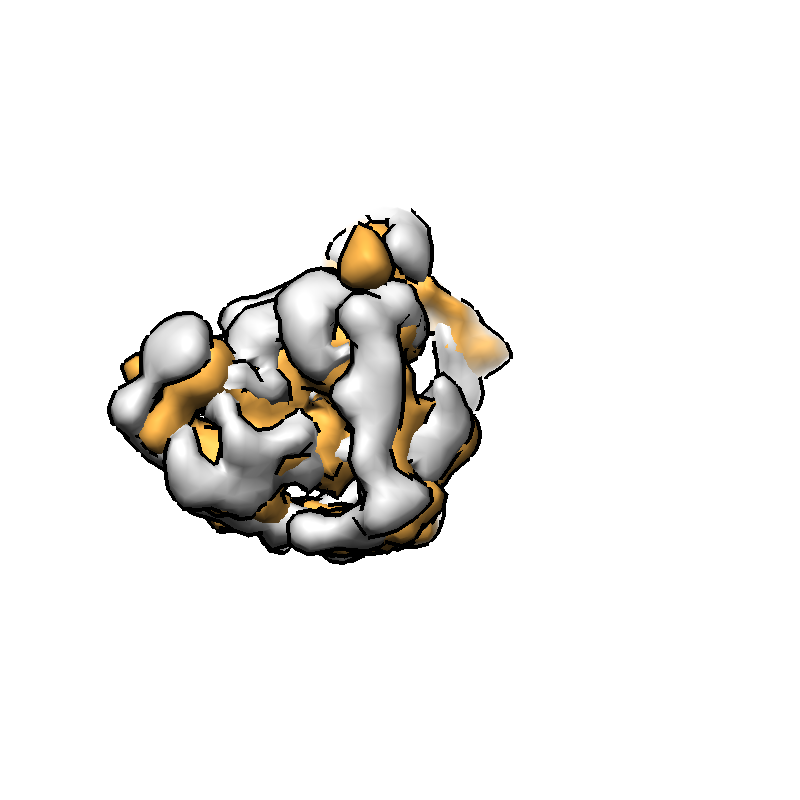

Supplement: Figure 2—source data 1. [file elife-49806-fig2-data1.zip › Figure 2-source data/EM density/WTATPgS_k6al/s3_WA6b.png]

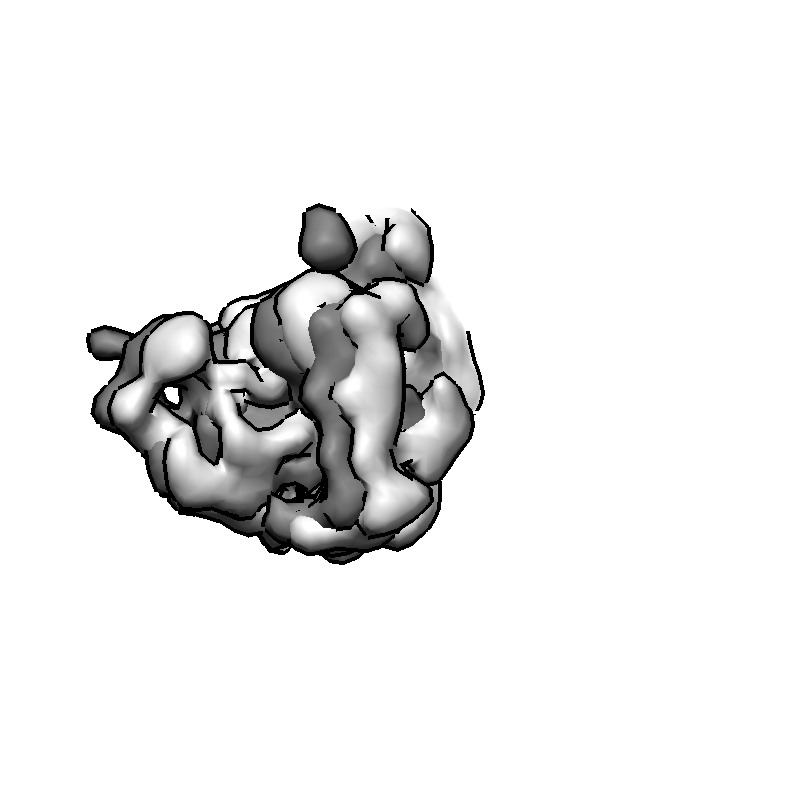

Supplement: Figure 2—source data 1. [file elife-49806-fig2-data1.zip › Figure 2-source data/EM density/WTATPgS_k6al/s2_WA6b.png]

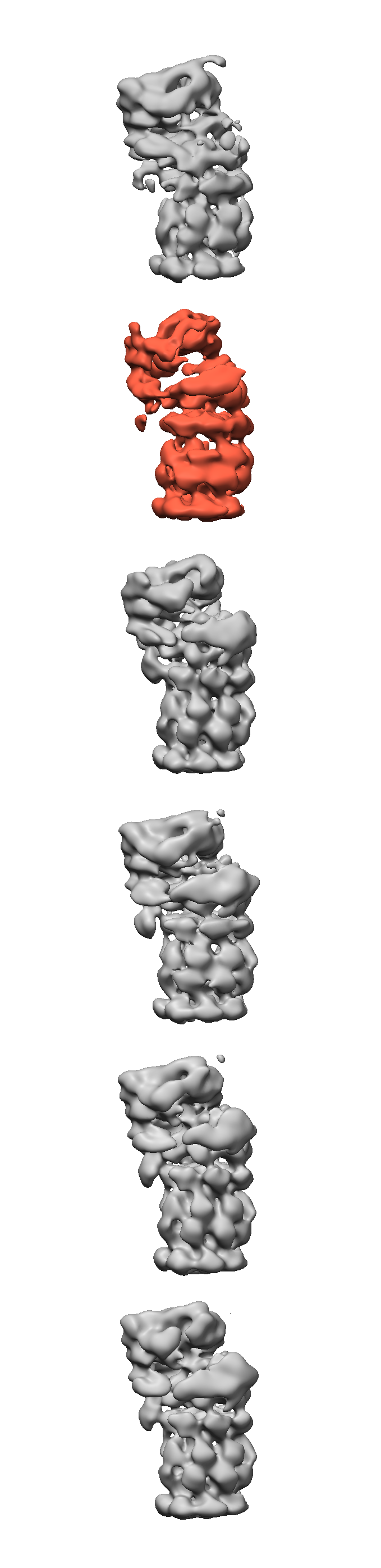

Supplement: Figure 2—source data 1. [file elife-49806-fig2-data1.zip › Figure 2-source data/EM density/WTATPgS_k6al/tiles_1to6.png]

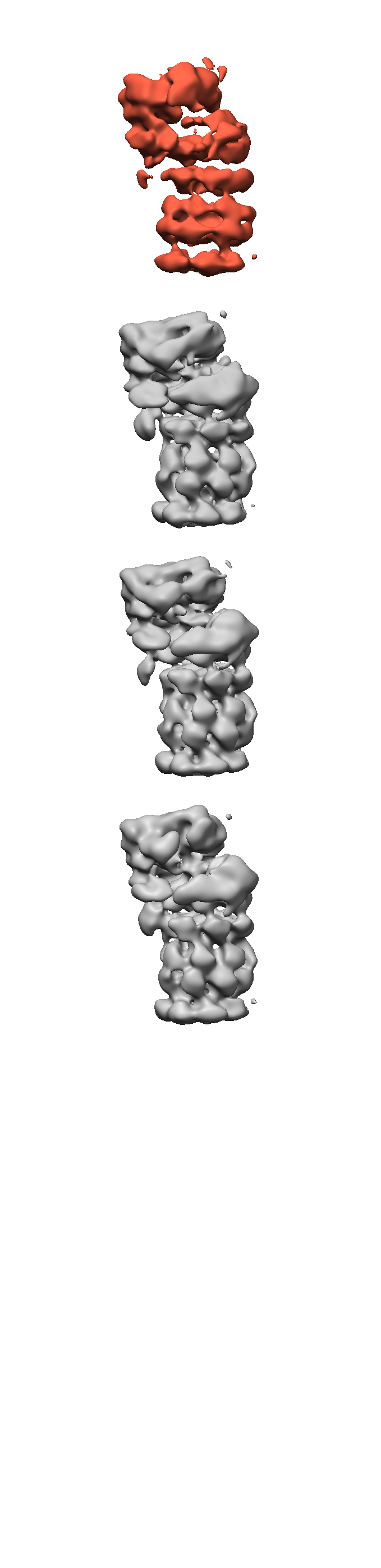

Supplement: Figure 2—source data 1. [file elife-49806-fig2-data1.zip › Figure 2-source data/EM density/WTATPgS_k6al/classes_c4k2_c6k2.png]

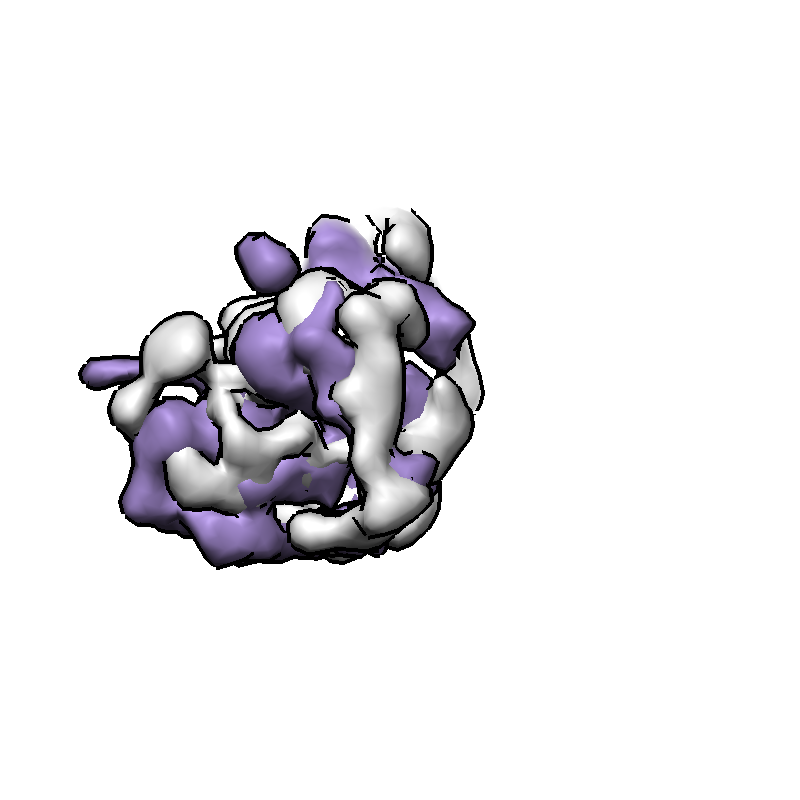

Supplement: Figure 2—source data 1. [file elife-49806-fig2-data1.zip › Figure 2-source data/EM density/WTATPgS_k6al/s1_WA6b.png]

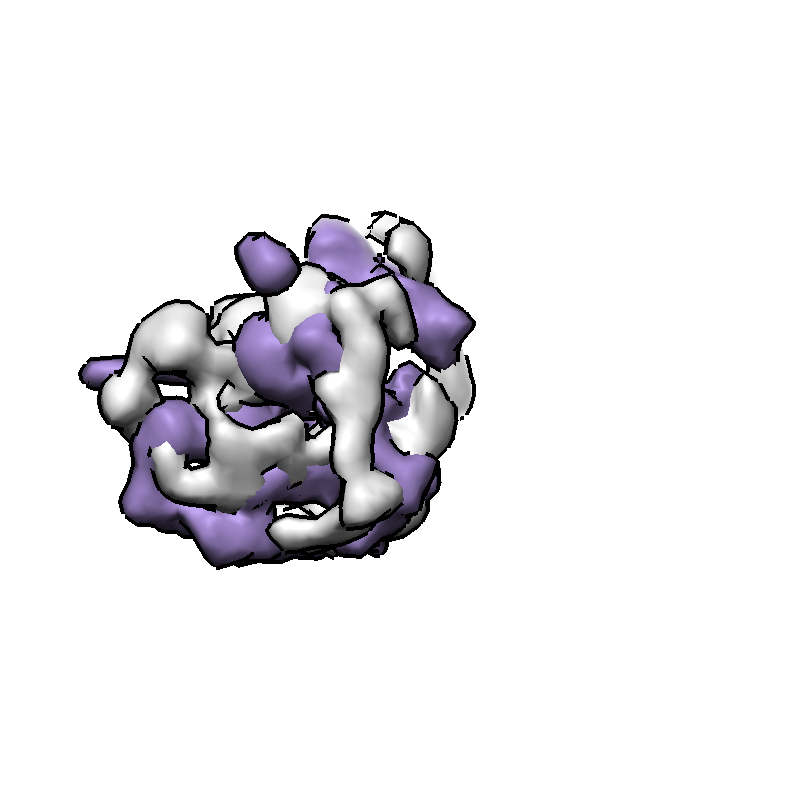

Supplement: Figure 2—source data 1. [file elife-49806-fig2-data1.zip › Figure 2-source data/EM density/WTATPgS_k6al/s1_WA6a.png]

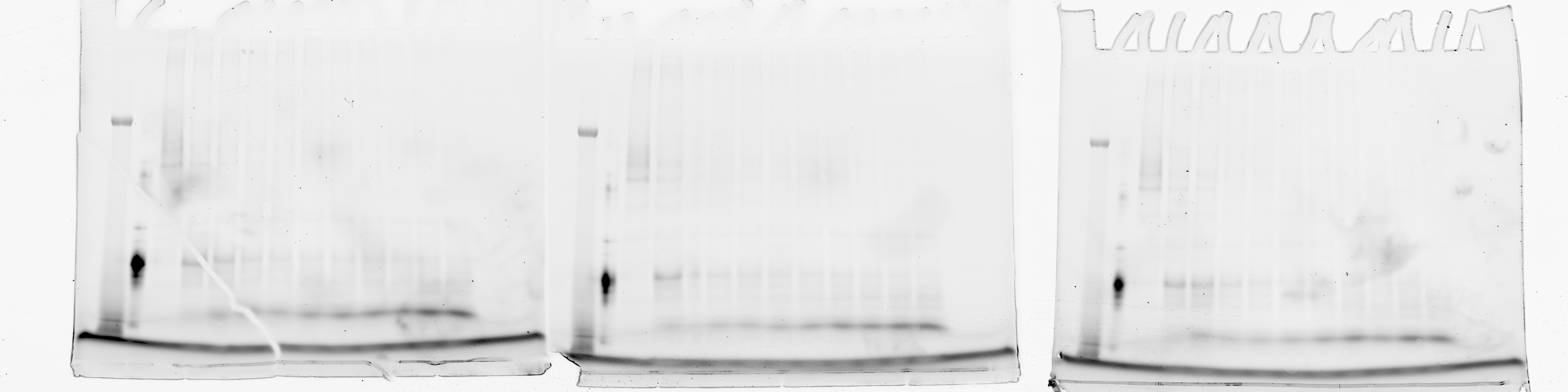

Supplement: Figure 5—figure supplement 2—source data 1. [file elife-49806-fig5-figsupp2-data1.zip › Figure5 Supplement 2B WT titin gel based degradation data/181228 wt titin eq6 26s 30c.tif]

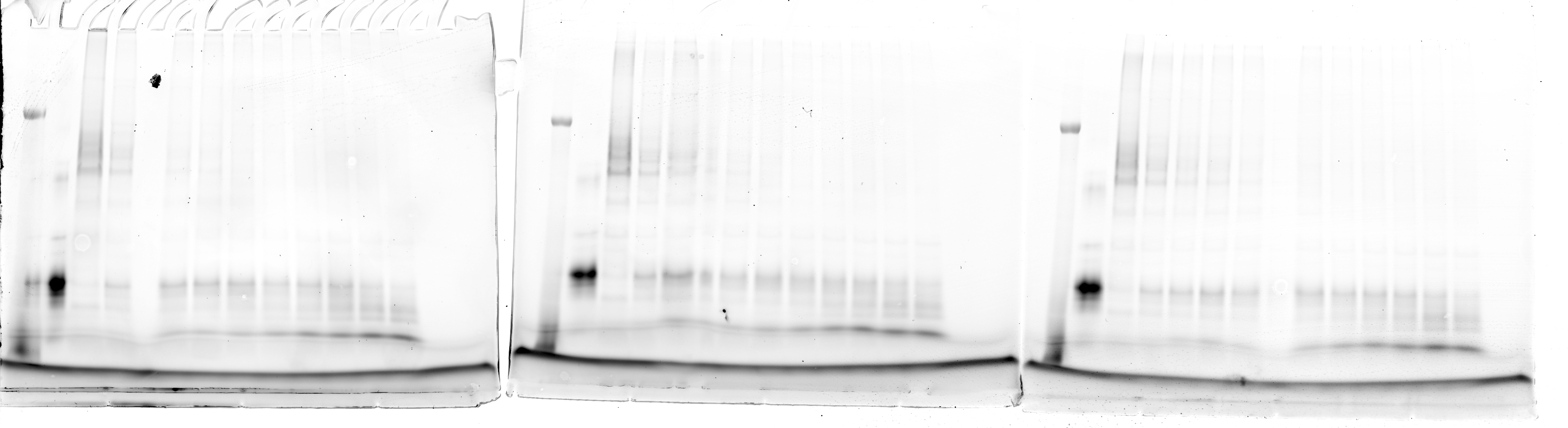

Supplement: Figure 5—figure supplement 2—source data 1. [file elife-49806-fig5-figsupp2-data1.zip › Figure5 Supplement 2B WT titin gel based degradation data/181228 wt titin vtenkif 26s 30c 3x.tif]

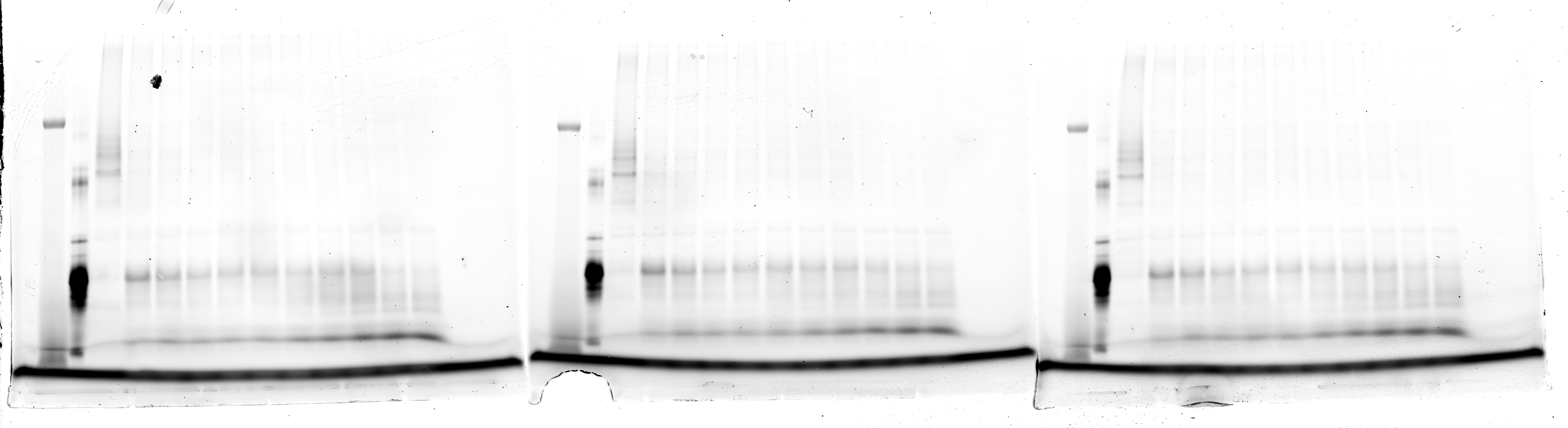

Supplement: Figure 5—figure supplement 2—source data 1. [file elife-49806-fig5-figsupp2-data1.zip › Figure5 Supplement 2B WT titin gel based degradation data/181228 wt titin wt 26s 30c 3x.tif]

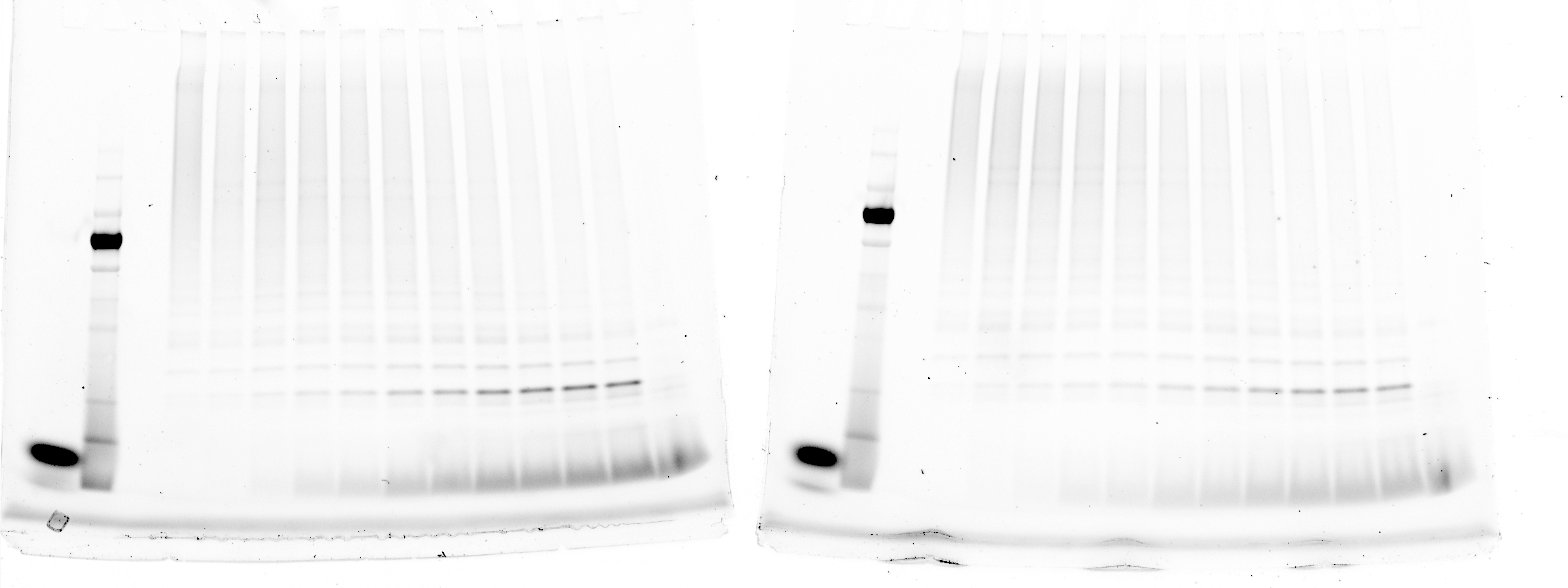

Supplement: Figure 5—figure supplement 2—source data 1. [file elife-49806-fig5-figsupp2-data1.zip › Figure5B and Figure5 Supplement 2A Restart Data/180503 vtenkif st1 2-[TAMRA] 50um.tif]

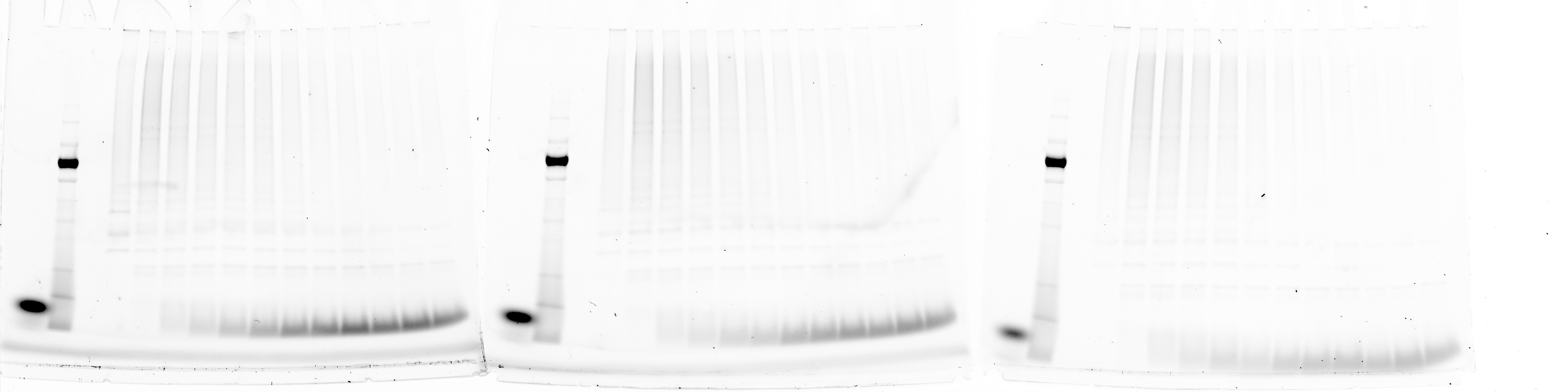

Supplement: Figure 5—figure supplement 2—source data 1. [file elife-49806-fig5-figsupp2-data1.zip › Figure5B and Figure5 Supplement 2A Restart Data/180504 wt st 1 2 3 .tif]

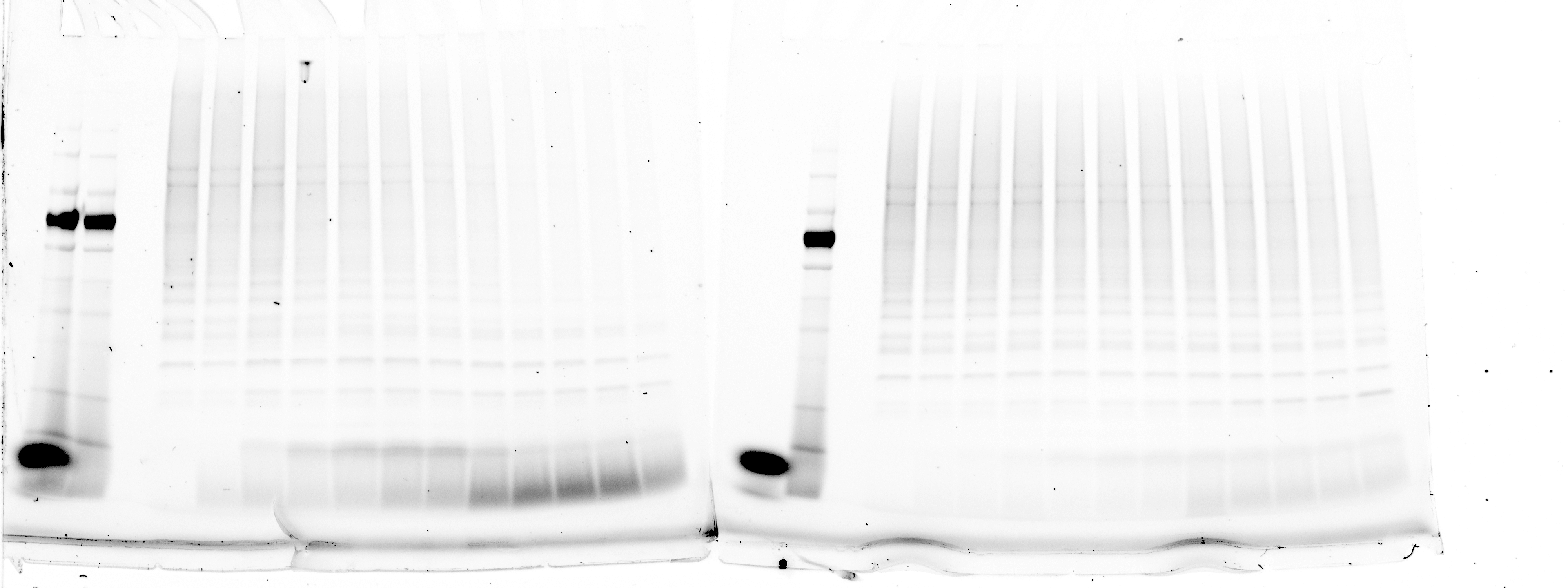

Supplement: Figure 5—figure supplement 2—source data 1. [file elife-49806-fig5-figsupp2-data1.zip › Figure5B and Figure5 Supplement 2A Restart Data/180515 wt restart 1 vtenkif restart 3.tif]

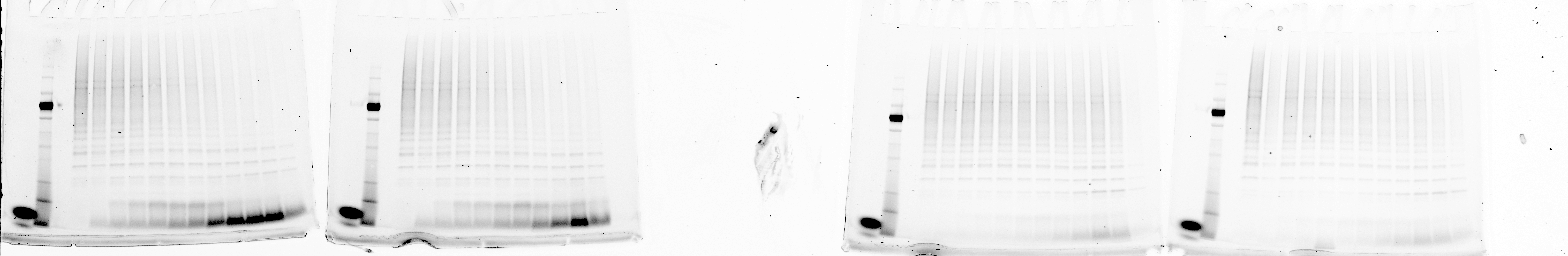

Supplement: Figure 5—figure supplement 2—source data 1. [file elife-49806-fig5-figsupp2-data1.zip › Figure5B and Figure5 Supplement 2A Restart Data/180515 wt restart 2 3 vtenkif restart 1 2-[TAMRA].tif]

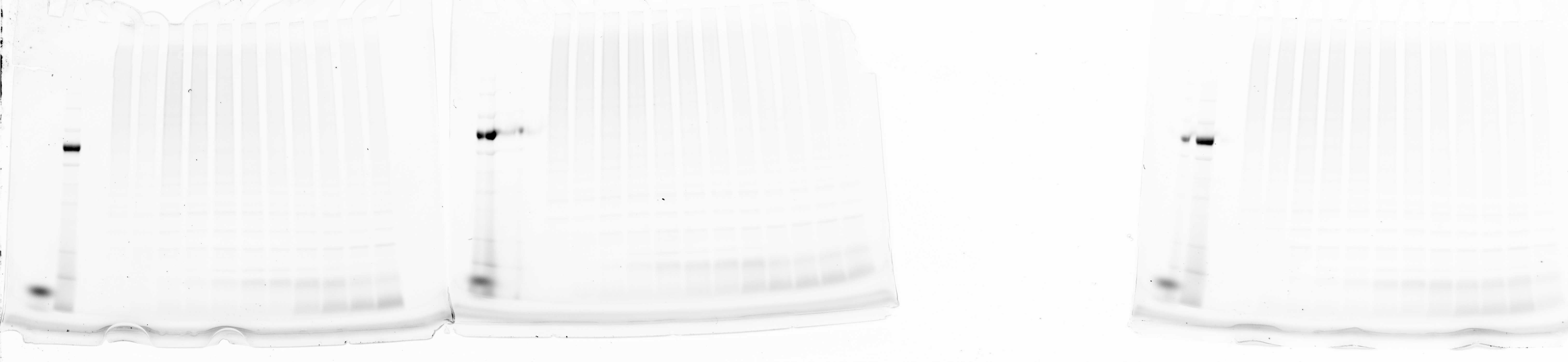

Supplement: Figure 5—figure supplement 2—source data 1. [file elife-49806-fig5-figsupp2-data1.zip › Figure5B and Figure5 Supplement 2A Restart Data/180611 eq6 rt.tif]

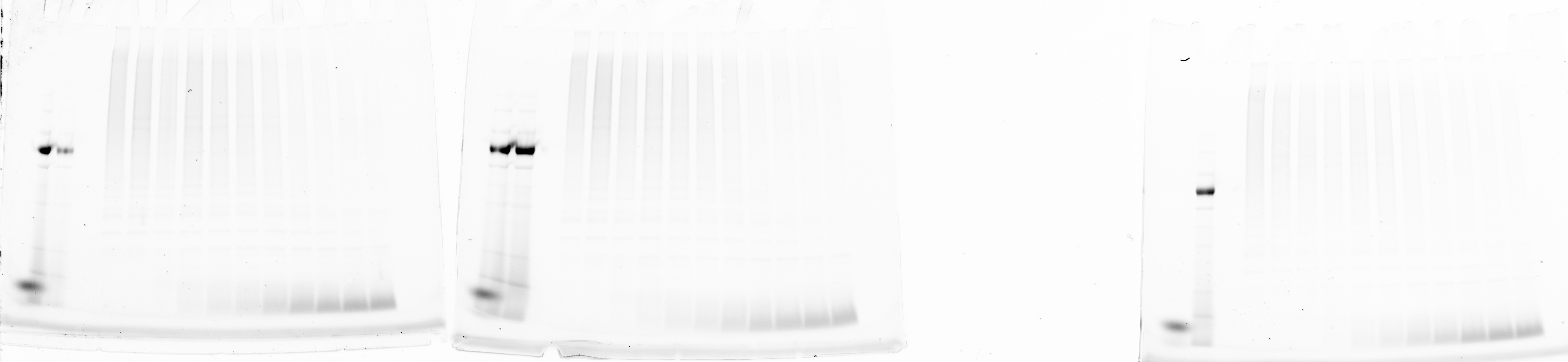

Supplement: Figure 5—figure supplement 2—source data 1. [file elife-49806-fig5-figsupp2-data1.zip › Figure5B and Figure5 Supplement 2A Restart Data/180611 eq6 st.tif]

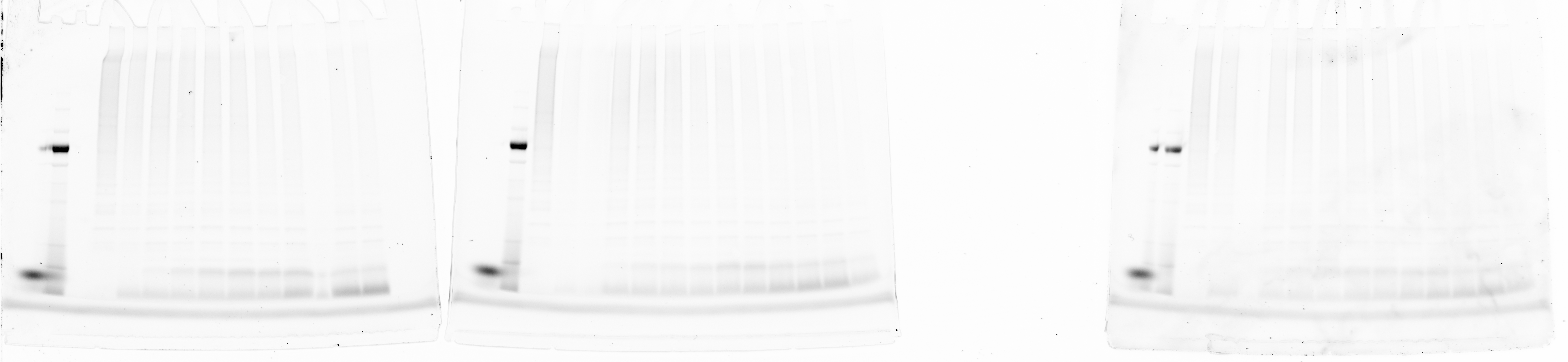

Supplement: Figure 5—figure supplement 2—source data 1. [file elife-49806-fig5-figsupp2-data1.zip › Figure5B and Figure5 Supplement 2A Restart Data/180611 wt rt.tif]

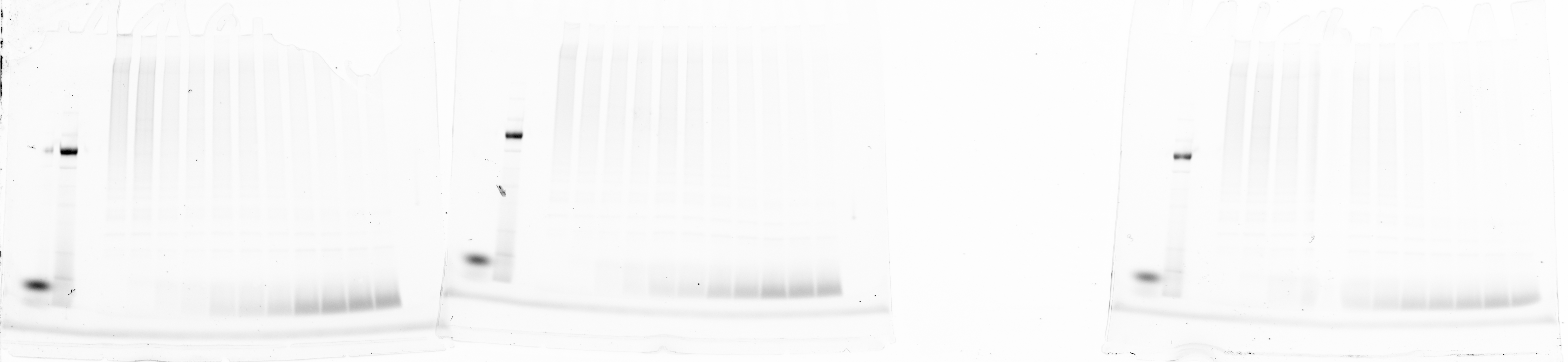

Supplement: Figure 5—figure supplement 2—source data 1. [file elife-49806-fig5-figsupp2-data1.zip › Figure5B and Figure5 Supplement 2A Restart Data/180611 wt st.tif]
